# Genomic, Epigenomic, and Transcriptomic Profiling towards Identifying Omics Features and Specific Biomarkers That Distinguish Uterine Leiomyosarcoma and Leiomyoma at Molecular Levels

**DOI:** 10.1155/2015/412068

**Published:** 2015-12-28

**Authors:** Tomoko Miyata, Kenzo Sonoda, Junko Tomikawa, Chiharu Tayama, Kohji Okamura, Kayoko Maehara, Hiroaki Kobayashi, Norio Wake, Kiyoko Kato, Kenichiro Hata, Kazuhiko Nakabayashi

**Affiliations:** ^1^Department of Maternal-Fetal Biology, National Research Institute for Child Health and Development, 2-10-1 Okura, Setagaya, Tokyo 157-8535, Japan; ^2^Department of Obstetrics and Gynecology, Graduate School of Medical Sciences, Kyushu University, 3-1-1 Maidashi, Higashi-ku, Fukuoka 812-8582, Japan; ^3^Department of Systems BioMedicine, National Research Institute for Child Health and Development, 2-10-1 Okura, Setagaya, Tokyo 157-8535, Japan; ^4^Department of Health Nutrition, Faculty of Health Science, Kio University, 4-2-4 Umami-naka, Koryo-cho, Kitakatsuragi-gun 635-0832, Japan; ^5^Department of Obstetrics and Gynecology, Faculty of Medicine, Kagoshima University, 8-35-1 Sakuragaoka, Kagoshima 890-0075, Japan

## Abstract

Uterine leiomyosarcoma (LMS) is the worst malignancy among the gynecologic cancers. Uterine leiomyoma (LM), a benign tumor of myometrial origin, is the most common among women of childbearing age. Because of their similar symptoms, it is difficult to preoperatively distinguish the two conditions only by ultrasound and pelvic MRI. While histopathological diagnosis is currently the main approach used to distinguish them postoperatively, unusual histologic variants of LM tend to be misdiagnosed as LMS. Therefore, development of molecular diagnosis as an alternative or confirmatory means will help to diagnose LMS more accurately. We adopted omics-based technologies to identify genome-wide features to distinguish LMS from LM and revealed that copy number, gene expression, and DNA methylation profiles successfully distinguished these tumors. LMS was found to possess features typically observed in malignant solid tumors, such as extensive chromosomal abnormalities, overexpression of cell cycle-related genes, hypomethylation spreading through large genomic regions, and frequent hypermethylation at the polycomb group target genes and protocadherin genes. We also identified candidate expression and DNA methylation markers, which will facilitate establishing postoperative molecular diagnostic tests based on conventional quantitative assays. Our results demonstrate the feasibility of establishing such tests and the possibility of developing preoperative and noninvasive methods.

## 1. Introduction

Uterine sarcoma is a malignant mesenchymal tumor composed of cells derived from uterine myometrium and represents the worst prognostic disease in gynecologic malignancies. The incidence of uterine sarcoma has been estimated to account for 8% of primary uterine malignancies [1]. Three major subtypes of uterine sarcomas are carcinosarcoma, endometrial stromal sarcoma, and leiomyosarcoma (LMS), all of which are resistant to surgery, chemotherapy, and radiotherapy. Although patients' prognosis is dependent on histopathological subtype and stage, 5-year relative survival rates of uterine sarcoma are 63–73%, 24–43%, 32–38%, and 6% at stages I, II, III, and IV, respectively, of the staging system determined by the International Federation of Gynecology and Obstetrics (FIGO) [2, 3]. LMS represents the most common subtype and mostly occurs in menopausal women over 40 years of age, who usually present symptoms such as abnormal vaginal bleeding, palpable pelvic mass, and pelvic pain. As these symptoms resemble those of the far more common uterine leiomyoma (LM), particularly degenerated LM, it is difficult to preoperatively distinguish LMS and LM by ultrasound and pelvic MRI [1]. A meta-analysis of 133 studies showed that the prevalence of occult LMS at surgery for presumed LM was estimated to be approximately 1 in 2000 [4]. Occult LMS cases tend to be discovered at their late stages, since they are often observed (as presumed leiomyoma) in outpatient clinics. Histopathological diagnosis after surgery is the only currently available means to distinguish the two conditions. However, some LM variants, such as the mitotically active type and LM with massive lymphoid infiltration, may be misdiagnosed as LMS during histopathological examination. In fact, in a previous population-based study of uterine sarcoma, of the 356 cases initially classified in the study as LMS, 97 cases (27%) were reclassified as LM or LM variants after review [5]. Introduction of confirmatory molecular diagnosis in addition to histopathological diagnosis is an option to be considered to decrease the risk of making inaccurate diagnoses. Identification of novel molecular markers highly specific to LMS will help to further improve the diagnostic accuracy of LMS.

“Cancer genomics” refers to the profiling of tumor genomes using various strategies such as DNA copy number, DNA methylation, transcriptome, and whole-genome sequencing [6]. Such omics data have been successfully utilized to identify genes and pathways perturbed in cancer and to discover novel diagnostic, prognostic, and therapeutic markers in several cancer types [7–9]. Such precedent and successful cases of cancer omics approaches at various phases suggest the necessity and importance of collecting omics datasets for gynecologic cancers as a basis towards identifying their diagnostic and prognostic markers and therapeutic targets. However, due to the rare incidence of LMS (0.4 in 100,000 [10]), the available omics profiles for LMS have been very limited so far. In the present study, we aimed at comprehensively understanding the differences between LM and LMS at the molecular level by analyzing the genomic, epigenomic, and transcriptomic profiles of these benign and malignant uterine tumors and also at extracting candidate expression and DNA methylation markers, which are potentially useful to establish molecular diagnostic tests using conventional assays.

## 2. Materials and Methods

### 2.1. Samples

We obtained three samples each of normal uterus myometrium tissue (NM1, NM2, and NM3), leiomyoma tissue (LM1, LM2, and LM3), and leiomyosarcoma tissue (LMS1, LMS2, and LMS3) from nine patients, whose clinical features are summarized in Table 1. Although NM samples were collected from cervical cancer patients, cancerous tissues were not mixed. Both LM and LMS samples were obtained from the center of the nidus. In three LMS cases, the LMS area was observed not to be associated with any LM-like areas by eye examination at surgery. The present study was approved by the Ethics Committee of the National Center for Child Health and Development (#234) and the Ethics Committee of Kyushu University (#231). All participating patients signed informed consent forms. All specimens were isolated during primary surgery at the Department of Obstetrics and Gynecology in the Kyushu University Hospital, immediately frozen in liquid nitrogen, and stored at −80°C. The cell lines examined in this study were SKN (RBRC-RCB0513) obtained from the RIKEN BRC and SK-UT1 (HTB-114) and SK-UT1B (HTB-115) obtained from the American Type Culture Collection.

### 2.2. Cell Culture

All cells were cultured under standard culture conditions (at 37°C, 5% CO_2_ in air) in a medium supplemented with 15% fetal bovine serum and Penicillin-Streptomycin-Glutamine Liquid (final 1x, GIBCO 10378-016). HamF12 medium was used for SKN cells and Eagle medium was used for SK-UT1 and SK-UT1B cells.

### 2.3. Genomic DNA and Total RNA Preparation

Genome DNA and total RNA were isolated from tissues and cells using the AllPrep Mini Kit (Qiagen) according to the manufacturer's protocol.

### 2.4. Single Nucleotide Polymorphism (SNP) Array Analysis for the Detection of Chromosomal Abnormalities

DNA amplification, labeling, and hybridization were performed according to the manufacturer's protocol of the HumanCytoSNP-12 BeadChip (Illumina). Two hundred ng of genomic DNA was subjected to DNA amplification for each of the samples. After hybridization and washing, the array slides were scanned on an iScan system (Illumina). Log *R* ratios (LRR) and B allele frequencies (BAF) were calculated using GenomeStudio version 2010.1 and visualized using KaryoStudio Data Analysis Software version 1.0 (Illumina). LRR and BAF represented the normalized signal intensity and the normalized ratio of the quantity of the B allele to the total quantity of both A and B alleles, respectively. Detection of copy neutral loss of heterozygosity (CNLOH) and copy number alterations (gain and loss) was performed using CNVpartition V3.0.7.0 (Illumina) and R-GADA (R-genome alteration detection analysis) [11], respectively, with default parameters.

### 2.5. Gene Expression Array Analysis

Total RNA samples were subjected to gene expression microarray analysis using the Whole Human Genome Microarray Kit, 4 × 44 K (Agilent), by following the manufacturer's instructions. This array contains a total of 41,093 probes covering 19,596 genes. Total RNAs (200 ng each) were amplified and labelled with Cy3 using the Low Input QuickAmp Labeling Kit (Agilent). The resultant cRNAs were fragmented at 60°C for 30 min in the dark, 600 ng of which was hybridized on the microarray at 65°C for 17 h. After washing, slides were then scanned using the Agilent microarray scanner G2505B. Signal intensities from the scanned images were determined using feature extraction software (version 10.7.3.1). Raw intensity data were then transferred to the GeneSpring software version 12.6 (Agilent), normalized (quantile normalization), and analyzed further using principal component analysis (PCA) and a selection of differentially expressed genes. The hierarchical clustering analysis for 1,036 differentially expressed genes was performed using Heatmap2 in the R package, gplots (https://cran.r-project.org/web/packages/gplots/gplots.pdf), with default parameters (complete linkage with Euclidean distance). The aforementioned 1,036 differentially expressed genes were selected as follows: Of 40,093 probes, 1,680 probes that were differentially expressed in at least one of the four groups of NM, LM, LMS, and LMS cell lines were selected by one-way ANOVA analysis (multiple testing correction, Benjamini-Hochberg; threshold, corrected *P* < 0.05) for log⁡2-transformed normalized signal intensity values; of these, 1,324 probes with a fold-change value of >2 or <0.5 in the group comparison between LMS and NM or LM and NM were further selected; of this number, 1,036 probes with a gene symbol were further selected (without redundancy of gene symbols). Gene ontology (GO) analysis was performed using the Database for Annotation, Visualization, and Integrated Discovery (DAVID) version 6.7 (https://david.ncifcrf.gov/). From the lists of probes ranked by fold-change values, the top 1,500 gene symbols were selected from up- and downregulated genes for each LM and LMS sample and subjected to GO analysis.

### 2.6. Genome-Wide DNA Methylation Analysis

Genomic DNA (1.5 *μ*g) was bisulfite converted using the EpiTect Plus DNA Bisulfite Kit (Qiagen). After determining the concentration of bisulfite-treated DNA, 300 ng of bisulfite DNA from each sample was subjected to Illumina Infinium HumanMethylation450 BeadChip analysis according to the manufacturer's standard protocol (Illumina). The array slides were scanned on an iScan system (Illumina). The scanned image data were processed using GenomeStudio Methylation Analysis Module version 1.9.0 with background subtraction and control normalization options. Methylation levels for each of over 480,000 CpG sites were calculated using a *β* value (= intensity of the methylated allele/[intensity of the unmethylated allele + intensity of the methylated allele + 100]), ranging from 0 (completely unmethylated) to 1 (completely methylated).

Of the 485,577 probes, probes with a detection *P* value of >0.05 or blank *β* value were excluded from further analyses. GenomeStudio was used to draw boxplots and scatter plots and to perform hierarchical clustering analysis (complete linkage with Euclidean distance). The hierarchical clustering analyses for the *β* values of subsets of hypermethylated probes were performed using Heatmap2 with default parameters. The difference in *β* values between the two samples was defined as delta beta (Δ*β*), and Δ*β* > 0.2 and Δ*β* < −0.2 were regarded as hyper- and hypomethylated, respectively, in this study. Differentially methylated regions between two groups of samples were extracted using Illumina Methylation Analyzer (IMA), an R package for analyzing site-level and region-level methylation changes between the two groups [12]. The BED-formatted lists of hyper- and hypomethylated regions were compiled for each of the six gene feature groups (TSS1500, TSS200, 5′UTR, first exon, gene body, and 3′UTR) by binning two or more probes found in the same gene feature group of the same gene as one region and analyzed one by one using the GREAT annotation website (http://bejerano.stanford.edu/great/public/html/) [13] with the default parameters of the “single nearest gene” mode.

### 2.7. Combined Bisulfite Restriction (COBRA) Analysis for LINE1

Bisulfite PCR for LINE1 elements were conducted as described previously [14]. The PCR primers used were 5′-TTGAGTTGTGGTGGGTTTATTTAG-3′ and 5′-TCATCTCACTAAAAAATACCAAACA-3′. The thermal cycling conditions were 25 cycles of 95°C for 30 seconds (s), 50°C for 30 s, and 72°C for 30 s, with an initial step of 95°C for 5 minutes (min) and a final step of 72°C for 2 min. PCR products (413 bp) were purified using the illustra GFX 96 PCR Purification Kit (GE Healthcare) and digested with* Hinf*I. The digestion DNA products were analyzed using BioAnalyzer 2100 (Agilent).

### 2.8. Data Deposition

DNA methylation and gene expression array data used in publication have been deposited in NCBI's Gene Expression Omnibu (http://www.ncbi.nlm.nih.gov/geo/) and are accessible through GEO Series accession number GSE68312.

## 3. Results and Discussion

### 3.1. Extensive Chromosomal Abnormalities Specific to Leiomyosarcoma

**Figure 1:**
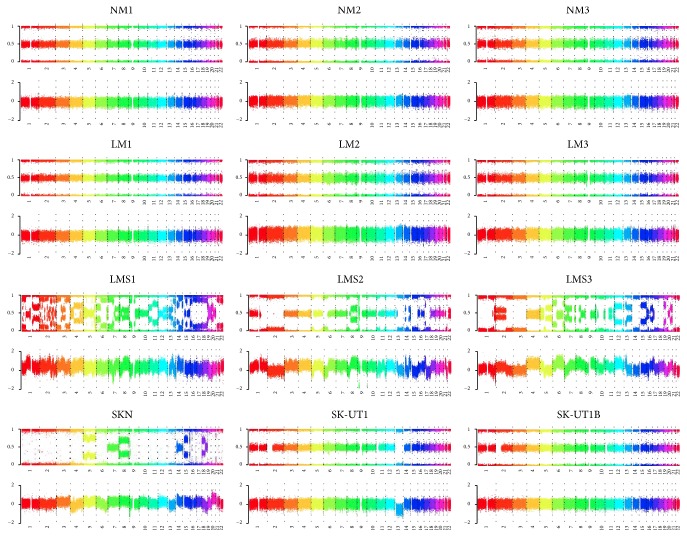
Chromosomal constitutions of normal metrium tissues (NM), leiomyoma (LM), and leiomyosarcoma (LMS) cases, and LMS-derived cell lines determined by SNP-array analysis. BAF (upper) and LRR (lower) are shown for 22 autosomal chromosomes of each of the 12 samples. The data ranges shown are 0 to 1 for BAF and −2 to 2 for LRR.

We assessed the extent of chromosomal copy number alterations in NM, LM, and LMS samples and LMS-derived cell lines using SNP BeadChip arrays carrying probes for approximately 300,000 SNPs. This array platform uses log⁡*R* ratio (LRR) and B allele frequency (BAF) as metrics to detect copy number changes. Although microarray-based comparative genomic hybridization (array-CGH) has been previously used to assess the chromosomal abnormalities of uterine LMS [15, 16], this is the first study to apply a SNP-array platform to analyze the genomic structural alterations of LMS together with LM. SNP-array platforms are advantageous over the array-CGH method in that the platforms can detect copy neutral loss of heterozygosity and mosaics in addition to copy number alterations. LMS samples showed highly disturbed LRR and BAF distributions compared to NM and LM samples, suggesting extensive numerical and structural changes as well as a highly mosaic constitution of chromosomes (Figure 1).

To estimate the extent of chromosomal aberrations for each sample, we used two copy number variation (CNV) calling tools, the CNV partition plug-in V3.0.7.0 (Illumina) and the R-GADA package [11]. We initially used the CNV partition, which detected copy neutral loss of heterozygosity (CN-LOH) accurately but failed to call gains and losses under mosaic chromosomal constitution. Therefore, we subsequently used R-GADA, which better detected gains and losses at chromosomally mosaic regions. We regarded the sum of the ratio of the copy number altering changes (gain and loss) detected by R-GADA and that of copy neutral changes (CN-LOH) detected by CNV partition as the approximate ratio of chromosomal abnormalities in this study. Consistent with the appearance of their LRR and BAF plots (Figure 1), LMS samples were highly chromosomally abnormal (66.7–89.5%) (Supplementary Material, Table S1, in Supplementary Material available online at http://dx.doi.org/10.1155/2015/412068). Compared to the LRR patterns of the LMS samples, those of the LMS-derived cell lines (SKN, SK-UT1, and SK-UT1B) were less complex, indicating that these cell lines are composed of cells with a single type or limited types of chromosomal constitution(s), which is likely due to a selection for cells capable of rapid and infinite cell division. LM samples were found to contain no or limited ratios of chromosomal abnormalities (0.7 to 8.7%). The numerical and structural abnormalities across almost all chromosomes are the genomic features specific to LMS, at least among the samples examined in this study, suggesting the possible use of CNV profiles as diagnostic information for LMS.

**Figure 2:**
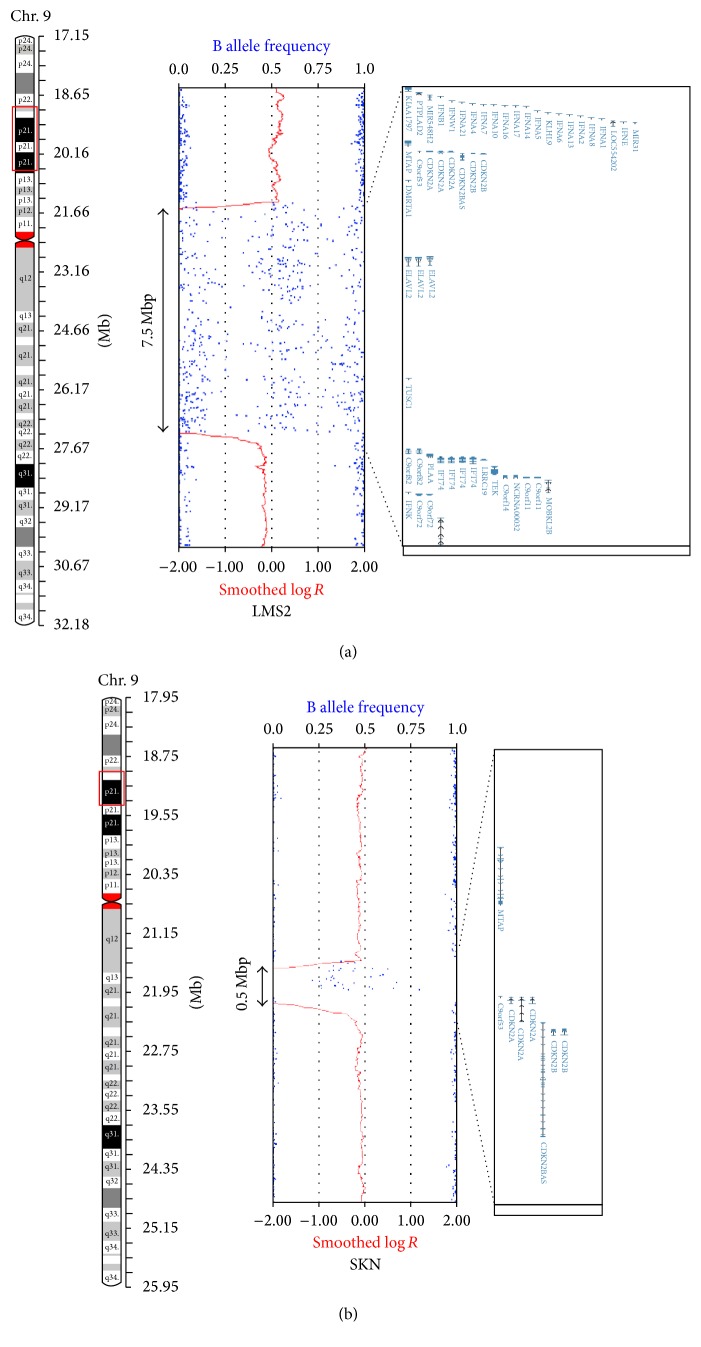
Homozygous deletions involving the* CDKN2A* and* CDKN2B* genes at chromosome 9p21.3 detected in LMS2 and SKN. A 7.5 Mb region (Chr9: 20,908,374–28,420,020, hg19) and a 0.5 Mb region (Chr9: 21,695,893-22,195,820, hg19) were found to be homozygously deleted in LMS2 (a) and in SKN (b), respectively.

We found that the* CDKN2A* locus was homozygously deleted in LMS2 and SKN (Figure 2). The p16 protein, encoded by the* CDKN2A* gene, plays a critical role in the regulation of the G1 cell cycle phase by inhibiting cyclin D and RB proteins and is known to be inactivated in many types of carcinomas and sarcomas [17]. A study of 77 LMS cases in soft tissues other than the uterus showed that decreased p16 expression correlates with promoter methylation of the* CDKN2A* gene and poor prognoses in patients [18]. Consistent with a previous study reporting the homozygous deletion of the* CDKN2A* locus in uterine LMS [19], our data further confirms the involvement of the functional loss of p16 during the development of uterine LMS.

Somatic mutations in the* FH* gene (encoding fumarate hydratase) [20] and in the* MED12* gene (encoding mediator complex subunit 12) [21] have been identified in 1.3% of sporadic and 70% of unselected uterine LMs, respectively. Approximately 40% to 50% of LMs are reported to contain cytogenetic rearrangements, such as those involving 12q15 or 6p21 and 7q deletions [22]. Furthermore, complex chromosomal rearrangements involving 7q,* COL4A5-COL4A6*,* HMGA2,* and* RAD51B* loci have been reported to be observed in a subset of LMs [23]. Therefore, not only mutations in specific genes but also chromosomal abnormalities are likely involved in genetic causes of LM and should be further explored. The accumulation of CNV profiles of unusual variants of LM (such as mitotically active cases) is urgently needed.

### 3.2. Gene Expression Signatures and Candidate Expression Markers Distinguishing LMS from LM

**Figure 3:**
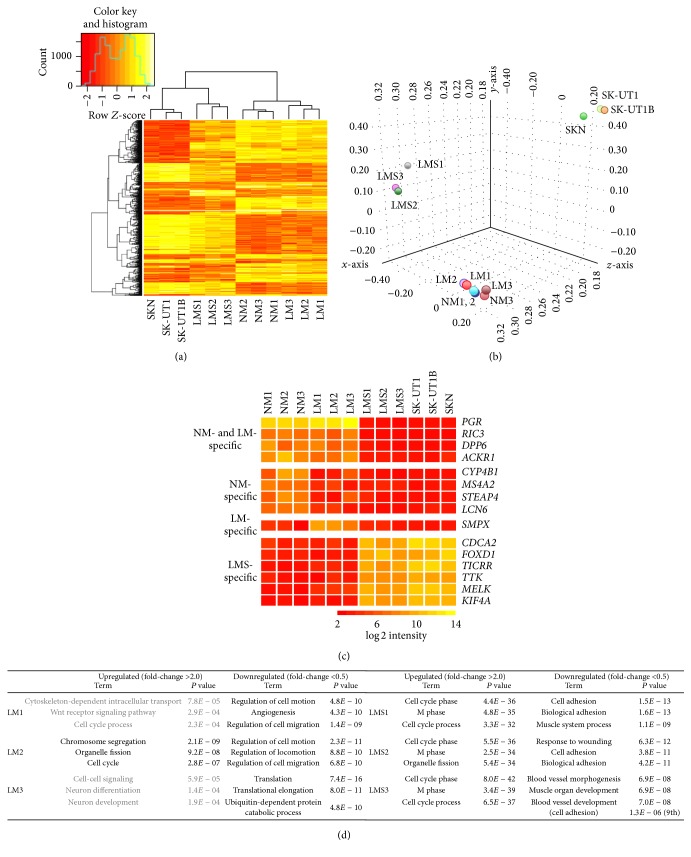
Transcriptome analysis for NM, LM, LMS, and LMS-derived cell lines. (a) Hierarchical clustering analysis for the normalized log⁡2-transformed intensities of the 1,036 differentially expressed genes using Heatmap2. (b) Three-dimensional visualization of PCA for the entire probe set. (c) Candidate expression markers. Of the 45 LMS-specific genes selected, only six, whose log⁡2 intensities in LMS and LMS-derived cell lines are >9, are shown (full genes are shown in Supplementary Material, Table S3). (d) GO analysis for up- and downregulated genes in each of the LM and LMS samples. The top three GO terms of biological process and their *P* values are shown in black when the corresponding Benjamini's corrected *P* value was <0.05 or are otherwise shown in grey.

We obtained gene expression profiles of NM, LM, and LMS samples together with those of LMS cell lines and found that hierarchical clustering for a subset of differentially expressed genes (1,036 genes selected as described in Section 2) and the PCA for the entire dataset reliably distinguished LMS samples (and LMS cell lines) from NM and LM samples (Figures 3(a) and 3(b)), as shown previously [24]. To search for candidate expression markers distinguishing NM, LM, and LMS, we selected probes whose signal intensities are high in one (or two) of the three types and are low (below the background level) in the other types. Using flag (present/absent call) and normalized log⁡2 intensity of “>6” as filters (Supplementary Material, Table S2), four NM/LM-specific, four NM-specific, one LM-specific, and 45 LMS-specific genes were selected (Figure 3(c) and Supplementary Material, Table S3). The selected LMS-specific genes were found to contain many genes known to encode a key cell cycle-related protein (such as* TICRR* [25] and* KIF4A* [26]) and to be involved in the cell cycle progression of cancerous cells (such as* CDCA2* [27] and* MELK* [28]). The 45 LMS-specific genes contain a number of critical cell cycle regulators and transcription factors that were not identified as genes overexpressed in LMS in a previous expression array study [24]. Therefore, the expression array dataset in this study provides additional information that facilitates understanding of the cancer biology of LMS. Although these candidate markers need to be validated in larger numbers of samples for their specificity, it is likely possible to establish a quantitative RT-PCR-based diagnostic method to distinguish LMS and LM using a combination of highly specific markers, for example, the* PGR* gene (encoding progesterone receptor) silenced in LMS and one of the LMS-specific cell cycle-related genes.

We counted the numbers of differentially expressed genes, upregulated (>2.0-fold) or downregulated (<0.5-fold), in three each of LM and LMS samples compared to the average of three NM samples (Supplementary Material, Figure S1). Although the numbers of upregulated and downregulated genes in six samples were similar, ranging from 5,999 to 8,932 and from 3,473 to 6,155, respectively (Supplementary Material, Figure S1), the numbers of probes commonly up- or downregulated among three samples differed significantly. In the three LMS samples, 20.4% and 22.1% of probes were commonly up- and downregulated, respectively, whereas only 2.9% and 4.7% were commonly up- and downregulated among the three LM samples (Supplementary Material, Figure S1), indicating that the contents of differentially expressed genes were similar among LMS samples but diverse among LM samples.

We performed GO analysis (see Section 2) to elucidate functional features of differentially expressed genes in LM and LMS samples compared to NM tissues. The results for the GOTERM_BP_FAT category (BP, biological process) were summarized in Figure 3(d) **(**full results are provided in Supplementary Material, Table S4). Commonly in all three LMS samples, genes related to the “cell cycle phase” and “cell adhesion” were statistically significantly enriched among up- and downregulated genes, respectively. In contrast, enrichment of GO terms differed across the three LM samples. Statistically significant (Benjamini's corrected *P* value < 0.05) enrichment of GO terms among upregulated genes was observed only in LM2 (“chromosome segregation”). Although the GO terms enriched among downregulated genes were very similar between LM1 and LM2 (“regulation of cell motion”), those in LM3 were totally different (“translation”). The observed downregulation of translation-related genes including many ribosomal protein genes and translation initiation/elongation factor genes was considered to be associated with the administration of the gonadotrophin-releasing hormone analogue to the patient of LM3 (Table 1).

Taken together, our annotations for the gene expression profiles of LM and LMS confirmed that these benign and malignant tumors can be distinguished by transcriptomic features as well as specific expression markers. However, as the three LM samples examined showed considerable variations in the contents of differentially expressed genes, data should be interpreted with caution. This also highlights the need for a dataset with a larger number of LM cases that can be used to define and classify the gene expression variations among the spectrum of LM cases.

### 3.3. Characterization of Genome-Wide DNA Methylation Profiles of LMS

**Figure 4:**
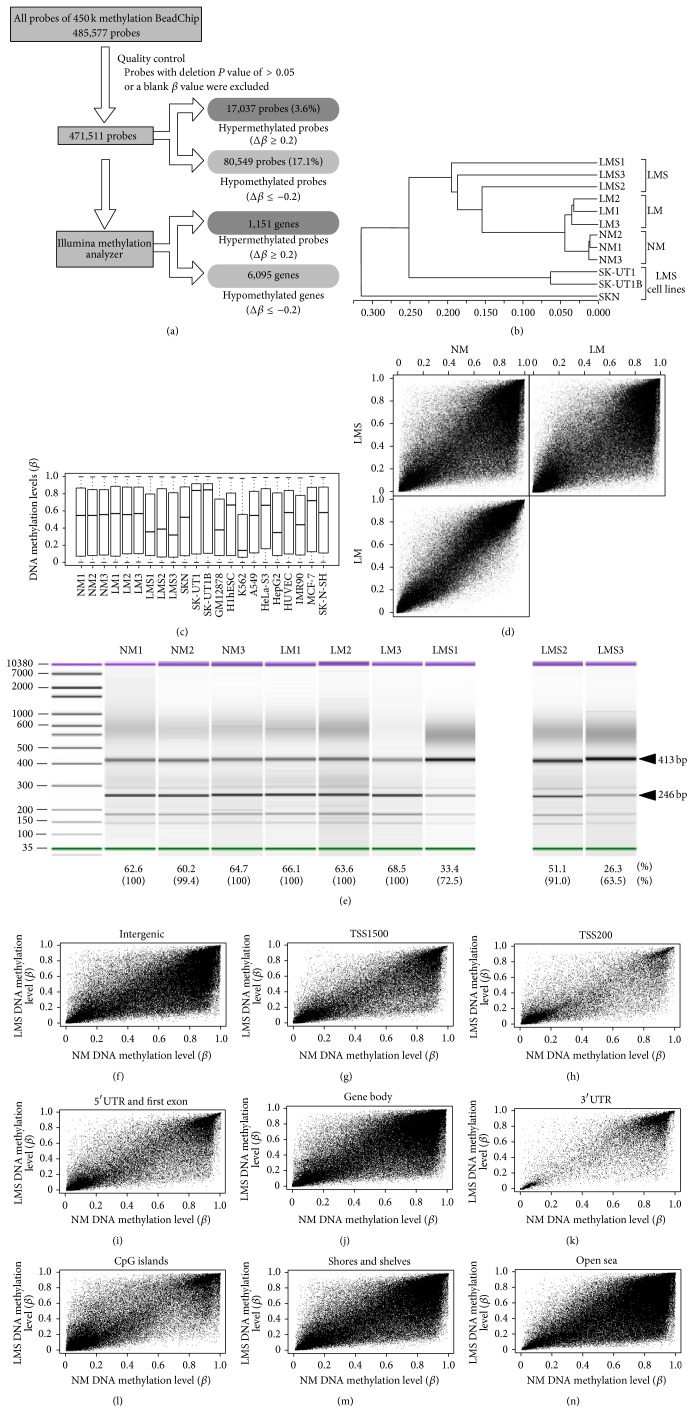
Comparisons and characterization of DNA methylation profiles of NM, LM, LMS, and LMS cell lines. (a) Flow chart showing results of quality control procedures and the extraction of differentially methylated probes and genomic regions. (b) Hierarchical clustering of 12 samples using *β* values of 471,511 probes that passed the data QC procedures (see (a)). (c) Boxplots of *β* values for the 471,511 probes that passed QC procedures for 12 samples analyzed in this study, as well as boxplots of the entire *β* values of HumanMethylation450 BeadChip data for ten cell lines obtained from the ENCODE DNA Methylation Track (http://hgdownload.cse.ucsc.edu/goldenPath/hg19/encodeDCC/wgEncodeHaibMethyl450/). Whereas LMS tissues exhibited global hypomethylation tendencies, LMS-derived cell lines did not. To assess whether observed global methylation levels (normal or hyper) are specific to LMS-derived cell lines, we examined those of six cancer cell lines (K562, A549, HeLa-S3, HepG2, MCF-7, and SK-N-SH) and confirmed their highly various levels. The DNA methylation profiles of these cancer cell lines as well as LMS-derived cell lines are likely extensively deviated from those of their origin (a cancerous tissue). (d) Scatter (*x*-*y*) plots showing the average *β* values of each sample for the 471,511 probes (NM versus LM, NM versus LMS, and LM versus LMS). (e) COBRA assays for LINE1 methylation. 413-bp and 246-bp bands represent uncut (unmethylated) and cut (methylated) bands upon* Hinf*I digestion, respectively. The methylation index (%) was calculated as (the intensity of the cut band/246)/((the intensity of the cut band/246) + (the intensity of the uncut band/413)) and shown at the bottom. The measured methylation index was corrected using the standard curve (Supplementary Material, Figure S2) obtained with the methylated and unmethylated control bisulfite-converted DNAs (EpiTect PCR Control DNA Set #59695, Qiagen). The corrected methylation levels (%) are shown in parentheses. Though the LINE1 methylation levels of LM were similar to those of NM, LMS samples showed lower levels of LINE1 methylation. (f)–(k) Scatter plots of *β* values (LMS average (*y*-axis) versus NM average (*x*-axis)) in the six gene feature groups. (l)–(n) Scatter plots of *β* values (LMS average (*y*-axis) versus NM average (*x*-axis)) of three subgroups in relation to CpG islands (CGIs): CGIs (l), CGI shores and shelves (within 4 kb distance from a CGI) (m), and non-CGI regions (over 4 kb distance from a CGI, open sea) (n). CpG sites within CGIs, the majority of which are unmethylated, were found to be more frequently hypermethylated (4.5%) than those outside of CGIs (2.5% in open sea) and to be much less frequently hypomethylated (4.6%) than those outside of CGIs (16.7% in shores and shelves, and 28.1% in open sea) (Table 2).

We obtained genome-wide DNA methylation profiles of NM, LM, and LMS samples and LMS-derived cell lines and used *β* values (DNA methylation levels) of 471,511 probes that passed quality control procedures (Figure 4(a)) for subsequent analyses. It should be noted that this is the first study describing the genome-wide DNA methylation profiles of LMS and LMS-derived cell lines. In a hierarchical clustering analysis, four groups (three histological types and the group of cell lines) were branched into different clusters (Figure 4(b)), clearly indicating that genome-wide DNA methylation datasets can distinguish NM, LM, and LMS. Boxplot representation (Figure 4(c)) of *β* values for all 471,511 probes revealed that the medians of *β* values in LMS samples were apparently lower compared to those of NM and LM. Scatter plots (Figure 4(d)) of the average *β* values for the same probe set also showed that LMS was globally hypomethylated compared to LM and NM, which is consistent with the global hypomethylation known to occur in most malignant tumors [29]. We confirmed the hypomethylation of LINE1 elements in LMS by the combined bisulfite restriction analysis (Figure 4(e) and Supplementary Material, Figure S2). These results demonstrate that LM and LMS can be distinguished by their global DNA methylation levels.

Since the genome-wide DNA methylation patterns of LM have been described previously [30], we focused on those of LMS in this study. To explore genomic features of hyper- and hypomethylation observed in LMS, we compared the average *β* values of LMS and NM samples in six gene feature categories, namely, intergenic, TSS1500 (200 bp to 1500 bp upstream from a transcription start site (TSS)), TSS200 (within 200 bp upstream from a TSS), 5′UTR (untranslated region) and the first exon, gene body, and 3′UTR (Figures 4(f)–4(k) and Table 2). The extent of hypomethylation was striking in the intergenic, TSS1500, gene body, and 3′UTR categories (29.0%, 14.4%, 15.8%, and 14.4%, resp.) (Figures 4(f), 4(g), 4(j), and 4(k)) but less striking in the TSS200 and 5′UTR/first-exon categories (7.2% and 10.0%) (Figures 4(h) and 4(i)). The extent of hypermethylation was consistently lower than that of hypomethylation in all six categories and tended to be lower in the TSS and its vicinities (2.1% to 3.2%) compared to other categories (3.9% to 4.5%). CpG sites within CpG islands (CGIs), the majority of which are unmethylated, were found to be more frequently hypermethylated (4.5%) than those outside of CGIs (2.5% in open sea) and to be much less frequently hypomethylated (4.6%) than those outside CGIs (16.7% in shores and shelves, and 28.1% in open sea) (Figures 4(l)–4(n) and Table 2). These results demonstrate that TSS and/or CGI regions tended to be resistant to genome-wide demethylation and were partly methylated* de novo*, during tumorigenesis.

When averages of the *β* values were compared between LMS and NM groups, 17,037 (3.6%) and 80,549 (17.1%) probes were hyper- (Δ*β* > 0.2) and hypomethylated (Δ*β* < −0.2), respectively. We subjected the same dataset to the IMA [12]: 1,151 and 6,095 genes were found to host hyper- and hypomethylated regions in at least one of the six gene feature groups of TSS1500, TSS200, 5′UTR, first exon, gene body, and 3′UTR** (** Figure 4(a) and Supplementary Material, Table S5). In the same analyses conducted for LM compared to NM, 14,053 (3.0%) and 9,510 (2.0%) probes were found to be hyper- and hypomethylated (Table 2), and 869 and 770 genes were found to host hyper- and hypomethylated regions in at least one of the six gene feature groups (data not shown).

**Figure 5:**
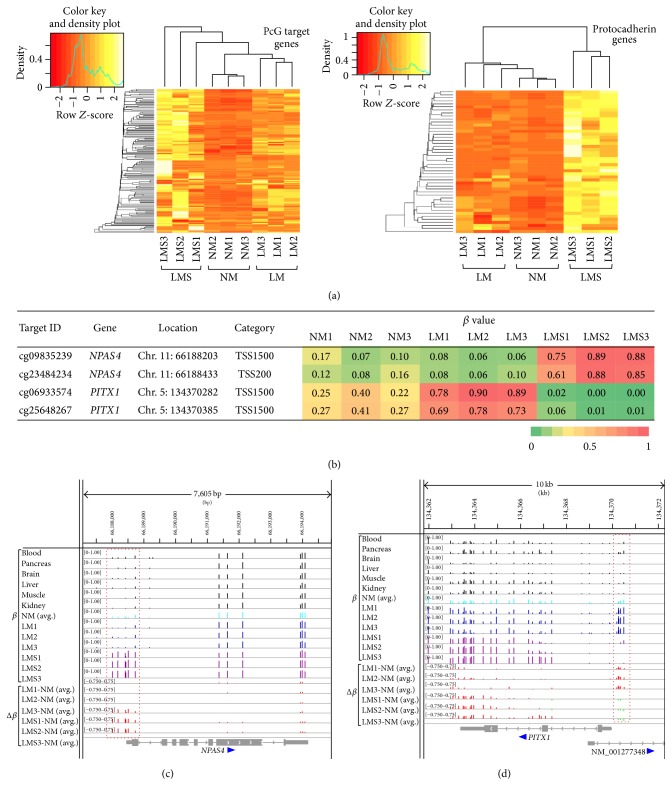
Candidate methylation markers to distinguish LM and LMS. (a) Clustering analyses of TSS200 probes at 37 PcG target gene loci (left) and 15 protocadherin gene loci (right) hypermethylated (Δ*β* > 0.2) in LMS compared to NM. The standard deviations of *β* values of 133 CpG probes (at 37 PcG target loci) ranged from 0.01 to 0.38 (median 0.22). The standard deviations of *β* values of 47 CpG probes (at 15 protocadherin genes) ranged from 0.06 to 0.31 (median 0.22). *β* values and *Z*-scores of those 133 and 47 CpG probes in NM, LM, and LMS samples are shown in Supplementary Material, Table S7. (b) Four selected promoter CpG sites whose methylation levels were strikingly different between LMS and LM. (c) and (d) DNA methylation profiles at* NPAS4* and* PITX1* loci. Methylation *β* values (data range: 0 to 1) and Δ*β* values (data range: −0.75 to 0.75) are shown as vertical bars using the Integrative Genomics Viewer. The colors of vertical bars for *β* values are as follows: black for six normal tissues (blood, pancreas, brain, liver, muscle, and kidney), light blue for the average of three NM samples (“NM(avg.)”), blue for LM samples, and purple for LMS samples. Positive and negative Δ*β* values for each of the LM and LMS samples (compared to NM(avg.)) are indicated by red and green vertical bars, respectively. The promoter region containing the probes shown in (b) is boxed by red dashed lines. Exon-intron structure and transcriptional orientation of the gene(s) are shown at the bottom.

We assessed the functional features of differentially methylated regions in LMS by GO analysis using the GREAT annotation website [13] as described in Section 2. We observed that hypermethylated regions were highly significantly enriched with homeobox and* PCDH* (protocadherin) genes (Supplementary Material, Table S5 and Figure S3). Polycomb group (PcG) target genes including many homeobox genes are known to be abnormally hypermethylated in various cancers [31]. Hypermethylation of the subset of PcG genes encoding developmental regulators is considered to potentially contribute to the stem-like state of cancer [32]. Protocadherin proteins contain extracellular cadherin domains involved in cell adhesion and are suggested to be candidate tumor suppressors because they modulate regulatory pathways (such as canonical Wnt signaling) that are critical in development and disease [33, 34]. Among 1,893 PcG target (SUZ12-positive) genes identified previously (Table S8 in [35]), 197 genes (10.4%) were found to be hypermethylated in LMS. Notably, 37 out of 197 genes were hypermethylated at the TSS200 region, which was found to be most resistant to hypermethylation (Figure 4(h)). Likewise, we identified 29 protocadherin genes to be hypermethylated in LMS, 15 of which were hypermethylated at TSS200. Hierarchical clustering of DNA methylation values of the aforementioned 37 PcG target genes and 15 protocadherin genes distinguished NM, LM, and LMS (Figure 5(a)).

A whole-genome bisulfite sequencing study has revealed that large blocks (up to several Mb) of hypomethylation were observed for more than half of the genome in colon cancers and that this feature was common in other solid tumors [36]. In the GO analysis, we observed that hypomethylated regions in LMS were frequently enriched with gene clusters such as olfactory receptor genes, kallikrein-related peptidase (*KLK*) genes, keratin-associated protein genes, and serine protease genes (Supplementary Material, Table S6 and Figure S4). These gene clusters were found to be located within genomic regions that were identified as hypomethylation blocks in colon cancers [36]. Taken together, the annotations for differentially methylated regions in LMS compared to NM demonstrate that LMS exhibits epigenomic features such as hypermethylation at the PcG target gene and protocadherin gene loci and hypomethylation within large blocks that are known to be common among malignant solid tumors (Supplementary Material, Figure S4).

We also assessed the relation between changes in the gene expression and the DNA methylation at gene promoter regions (TSS1500 and TSS200) observed in LMS compared to NM (Supplementary Material, Figure S5) and observed no correlation between them.

### 3.4. Selection of Candidate Methylation Markers to Distinguish LMS from LM

As an attempt to identify candidate DNA methylation marker loci to reliably distinguish LMS and LM, we selected 69 probes whose methylation levels were strikingly different between LMS and LM using *β* value > 0.6 in all three samples in one group and *β* value < 0.1 in all three samples in the other group as filter conditions (data not shown; 69 probes consisted of 13 probes and 56 probes hypermethylated in LMS and LM, resp.). Among the 69 probes, 38 probes were mapped to 31 gene loci (the remaining 31 probes were located intergenically). These 38 probes were further selected using the following conditions: two or more probes were mapped within the TSS1500/TSS200 categories of one gene. As a result, the promoter regions of* NPAS4* and* PITX1* genes were selected as examples of candidate methylation marker loci (Figures 5(b)–5(d)). It should be noted that different candidates can be selected by changing filter conditions. The promoter region of the* NPAS4* gene (encoding the neuronal PAS domain Protein 4) was nearly unmethylated in normal tissues and LM samples but highly methylated only in LMS samples (Figure 5(c)). The promoter region of the* PITX1* gene (encoding the paired-like homeodomain 1 protein) was highly methylated only in LM samples but not in normal tissues and LMS samples (Figure 5(d)).

### 3.5. Feasibility of Developing Molecular Diagnostic Tests to Distinguish LMS from LM

We successfully identified omics features as well as candidate biomarkers that can distinguish LMS from LM. The array-based methods used in this study require only a few hundred nanograms of genomic DNA or total RNA. Therefore, omics profiles can be obtained for limited amounts of specimens such as those collected by transcervical needle biopsy. There is also the option to introduce next-generation sequencing-based methods, which require even smaller amounts of starting materials. Recently, a method to capture sarcoma cells circulating in peripheral blood has been developed [37]. Combining such a method with LMS-specific markers or omics signatures identified in this study will open up the possibility to develop a preoperative and noninvasive diagnostic test to distinguish LM and LMS.

## 4. Conclusions

We have demonstrated that omics profiles clearly distinguish typical uterine LMS and LM and represent reservoirs for molecular markers highly specific to LMS. While the numbers of the samples assessed were limited in this study, the array platforms adopted and the data analysis methods established in this study are directly applicable to a larger number of samples. In clinical settings, there is an urgent need for gynecologic oncologists to establish reliable methodologies that distinguish the intermediate grades of tumors, such as uterine smooth muscle tumors of uncertain malignant potential and atypical LM, from LMS. By obtaining omics profiles for such intermediate grades of tumors, it could be plausible to screen for good molecular markers for these tumors using the dataset in this study as a reference. The omics profiling methods and the dataset described in this study could help to develop preoperative and noninvasive diagnostic tests for LMS.

## Supplementary Material

Figure S1: Venn diagrams for the probes detecting differential gene expression in LM and LMS samples compared to the average of NM samples.
Figure S2: A standard curve for the COBRA assay for LINE1 repetitive sequences.
Figure S3: Ontology terms in the InterPro category detected to be enriched among the differentially methylated regions in LMS (compared to NM) in the GREAT annotation.
Figure S4: DNA methylation profiles of NM, LM, and LMS samples visualized using Integrative Genomic Viewer (https://www.broadinstitute.org/igv/).
Figure S5: Methylation by expression plots of LMS compared to NM.
Table S1: Genomic sizes and ratios of the chromosomal abnormalities detected by SNP array analysis.
Table S2: Filter conditions used for the selection of candidate expression markers.
Table S3: Full list of candidate expression markers selected using filter conditions shown in Table S3.
Table S4: Full results of DAVID's gene ontology analysis for differentially expressed genes in each of LM and LMS samples compared to the average of NM samples.
Table S5: List of 7110 genes hosting differentially methylated regions in LMS compared to NM detected by IMA.
Table S6: Full results of GREAT annotation for differentially methylated regions in LMS compared to NM.
Table S7: β values and Z-scores of 133 CpG probes in the TSS200 regions of the 37 PcG target gene loci and of 47 CpG probes in the TSS200 regions of the 15 protocadherin gene loci hypermethylated (Δβ > 0.2) in LMS compared to NM.

## Figures and Tables

**Table 1:** Clinical features of nine samples analyzed in this study.

Sample	Clinical diagnosis	Tissue-type obtained	Age	Major axis of tumor (cm)	Preoperative therapy
NM1	Cervical cancer, stage Ib1	Normal myometrium	43	—	None
NM2	Cervical cancer, stage Ib2	Normal myometrium	38	—	None
NM3	Cervical cancer, stage Ib2	Normal myometrium	49	—	None
LM1	Interstitial myoma	Leiomyoma	52	18	None
LM2	Submucosal myoma	Leiomyoma	48	6	None
LM3	Submucosal myoma	Leiomyoma	53	6	GnRHa
LMS1	Leiomyosarcoma, stage IV	Leiomyosarcoma	49	25	None
LMS2	Leiomyosarcoma, stage I	Leiomyosarcoma	54	20	None
LMS3	Leiomyosarcoma, stage I	Leiomyosarcoma	83	10	None

GnRHa, gonadotrophin-releasing hormone analogues.

**Table 2:** Numbers of differentially methylated CpG sites in LM and LMS compared to normal metrium (NM) tissues.

Feature	# of CpG probes	Leiomyoma (LM)		Leiomyosarcoma (LMS)	
		Hypermethylated (Δ*β* > 0.2)	Hypomethylated (Δ*β* < −0.2)	Hypermethylated (Δ*β* > 0.2)	Hypomethylated (Δ*β* < −0.2)
*Gene feature groups*					
Intergenic	115,382	4,910 (4.3%)	2,776 (2.4%)	4,533 (3.9%)	33,513 (29.0%)
TSS1500	67,542	1,841 (2.7%)	1,059 (1.6%)	1,614 (2.4%)	9,741 (14.4%)
TSS200	50,513	877 (1.7%)	415 (0.8%)	1,043 (2.1%)	3,625 (7.2%)
5′UTR and 1st exon	63,745	1,504 (2.4%)	913 (1.4%)	2,025 (3.2%)	6,383 (10.0%)
Gene body	157,160	4,539 (2.9%)	3,894 (2.5%)	7,072 (4.5%)	24,809 (15.8%)
3′UTR	17,170	382 (2.2%)	453 (2.6%)	750 (4.4%)	2,478 (14.4%)
Total	**471,512 **	**14,053 (3.0%)**	**9,510 (2.0%)**	**17,037 (3.6%)**	**80,549 (17.1%)**

*Relation to CGI*					
CGI	145,443	3,848 (2.6%)	884 (0.6%)	6,541 (4.5%)	6,612 (4.5%)
Shore and shelf	156,254	5,230 (3.3%)	2,960 (1.9%)	6,180 (4.0%)	26,231 (16.8%)
Open sea^*∗*^	169,815	4,975 (2.9%)	5,666 (3.3%)	4,316 (2.5%)	47,706 (28.1%)
Total	**471,512 **	**14,053 (3.0%)**	**9,510 (2.0%)**	**17,037 (3.6%)**	**80,549 (17.1%)**

^*∗*^Outside of island, shore, and shelf.

Δ*β* of LM: average of the *β* values of three LM samples − average of the *β* values of three NM samples.

Δ*β* of LMS: average of the *β* values of three LMS samples − average of the *β* values of three NM samples.
