# Supplementary material for: Genomic, Epigenomic, and Transcriptomic Profiling towards Identifying Omics Features and Specific Biomarkers That Distinguish Uterine Leiomyosarcoma and Leiomyoma at Molecular Levels
Source: Sarcoma. 2015 Dec 28;2015:412068. doi: 10.1155/2015/412068 (PMC4707342; doi:10.1155/2015/412068)
Supplement: Supplementary file 1 — Figure S1: Venn diagrams for the probes detecting differential gene expression in LM and LMS samples compared to the average of NM samples. Figure S2: A standard curve for the COBRA assay for LINE1 repetitive sequences. Figure S3: Ontology terms in the InterPro category detected to be enriched among the differentially methylated regions in LMS (compared to NM) in the GREAT annotation. Figure S4: DNA methylation profiles of NM, LM, and LMS samples visualized using Integrative Genomic Viewer (https://www.broadinstitute.org/igv/). Figure S5: Methylation by expression plots of LMS compared to NM. Table S1: Genomic sizes and ratios of the chromosomal abnormalities detected by SNP array analysis. Table S2: Filter conditions used for the selection of candidate expression markers. Table S3: Full list of candidate expression markers selected using filter conditions shown in Table S3. Table S4: Full results of DAVID's gene ontology analysis for differentially expressed genes in each of LM and LMS samples compared to the average of NM samples. Table S5: List of 7110 genes hosting differentially methylated regions in LMS compared to NM detected by IMA. Table S6: Full results of GREAT annotation for differentially methylated regions in LMS compared to NM. Table S7: β values and Z-scores of 133 CpG probes in the TSS200 regions of the 37 PcG target gene loci and of 47 CpG probes in the TSS200 regions of the 15 protocadherin gene loci hypermethylated (Δβ > 0.2) in LMS compared to NM. [file 412068.f1.pdf]

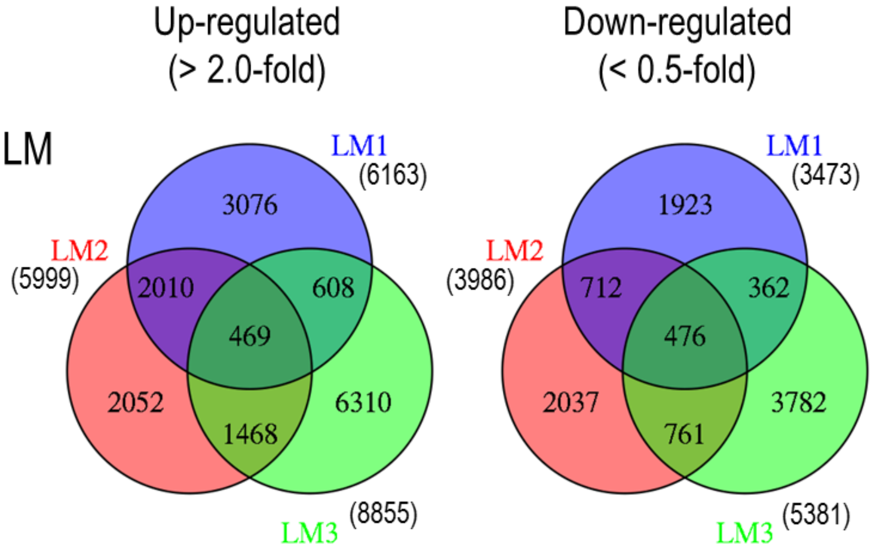

LM_up			
single	double	triple	total
11,438	4,086	469	15,993
71.5%	25.5%	2.9%	100.0%

LM_down			
single	double	triple	total
7,742	1,835	476	10,053
77.0%	18.3%	4.7%	100.0%

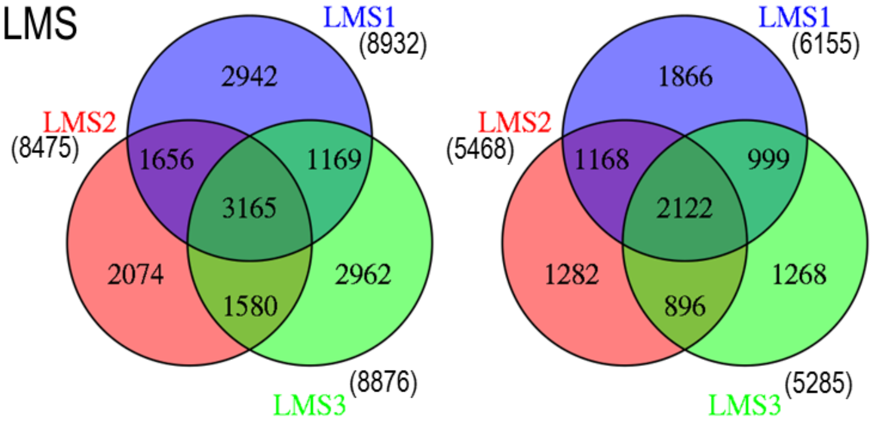

LMS_up			
single	double	triple	total
7,978	4,405	3,165	15,548
51.3%	28.3%	20.4%	100.0%

LMS_down			
single	double	triple	total
4,416	3,063	2,122	9,601
46.0%	31.9%	22.1%	100.0%

Supplementary Figure S1:
Venn diagrams for the probes detecting differential gene expression in LM and LMS samples compared to the average of NM samples.
Normalized log2-transformed intensity values were used to calculate fold changes between a sample (LM or LMS) and the average of three NM samples. To extract probes detecting up-regulation (> 2.0 fold), only probes whose present/absent call (gIsWellAboveBackground) is present in the LM or LMS sample were used. To extract probes detecting down-regulation (< 0.5 fold), only probes whose present/absent calls are all present in three NM samples sere used. The numbers (and ratios) of the probes detected in single, double, and triple samples were tabulized for each of the four categories (up- and down-regulated in LM and LMS samples).
Among 41,093 probes on the microarray, 6,163, 5,999 and 8,855 probes detected up-regulated (> 2.0-fold) gene expression, and 3,473, 3,986, and 5,381 probes detected down-regulated (< 0.5-fold) gene expression, in LM1, LM2, and LM3, respectively. In LMS1, LMS2, and LMS3, 8,932, 8,475, and 8,876 probes detected up-regulated gene expression, and 6,155, 5,468, and 5,285 probes detected down-regulated gene expression. In three LM samples, 469 and 476 probes commonly detected up- and down-regulation, respectively, which account for 2.9% and 4.7% of the total numbers of up-regulated and down-regulated probes (15,993 and 10,053, respectively). In three LMS samples, 3,165 and 2,122 probes commonly detected up- and down-regulation, respectively, which account for 20.4% and 22.1% of the total numbers of up-regulated and down-regulated probes (15,548 and 9,601, respectively).

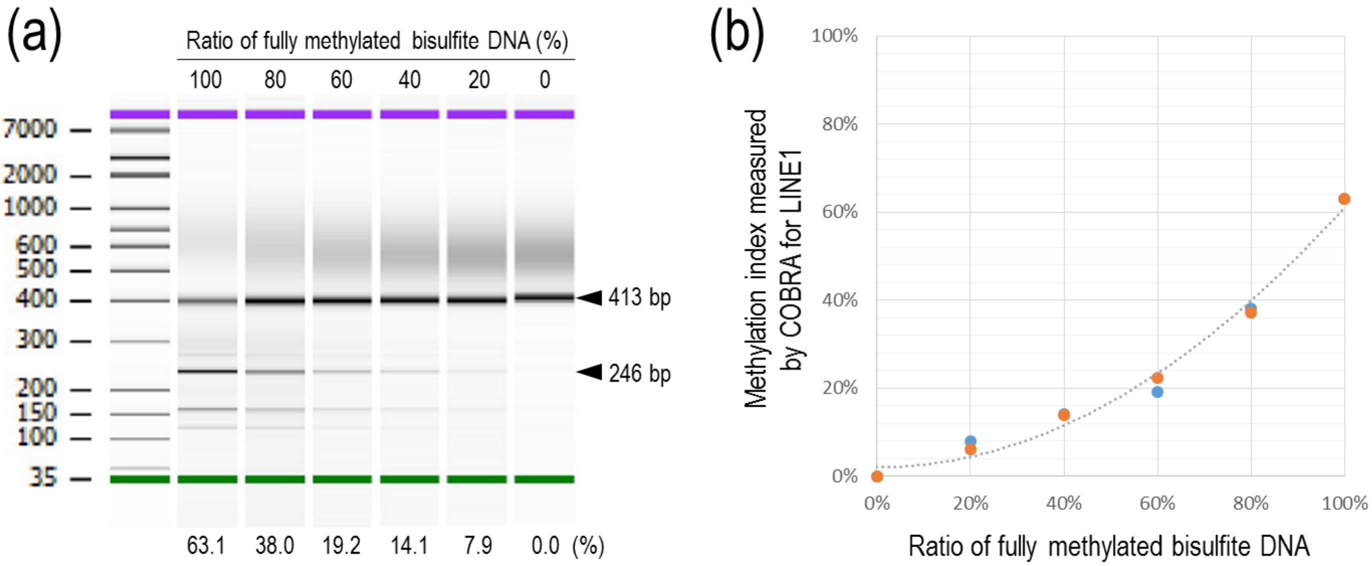

Supplementary Figure S2:
A standard curve for the COBRA assay for LINE1 repetitive sequences.
(a) Fully methylated and bisulfite-converted human control DNA, and unmethylated and bisulfite-converted human control DNA (EpiTect PCR Control DNA Set #59695, Qiagen) were mixed with the former ratio of 100%, 80%, 60%, 40%, 20%, and 0%, and subjected to COBRA assays for LINE1 sequences as described in the Materials and Methods. The methylation indexes (%) shown at the bottom were calculated as described in the legend of Figure 5 in the main text. 413-bp and 246-bp bands represent uncut (unmethylated) and cut (methylated) bands upon HinfI digestion, respectively. (b) A standard curve for the COBRA assay for LINE1 repetitive sequences. The aforementioned series of DNA mixtures were subjected to the COBRA assays for LINE1 in duplicate. X-axis, ratio of fully methylated bisulfite DNA; Y-axis, methylation index measured.

In the standard curve obtained, the methylation indexes measured were consistently lower than the ratios of fully methylated bisulfite-converted DNA. Loss of HinfI site due to DNA polymorphism in a subset of LINE1 copies are most likely responsible for this tendency.

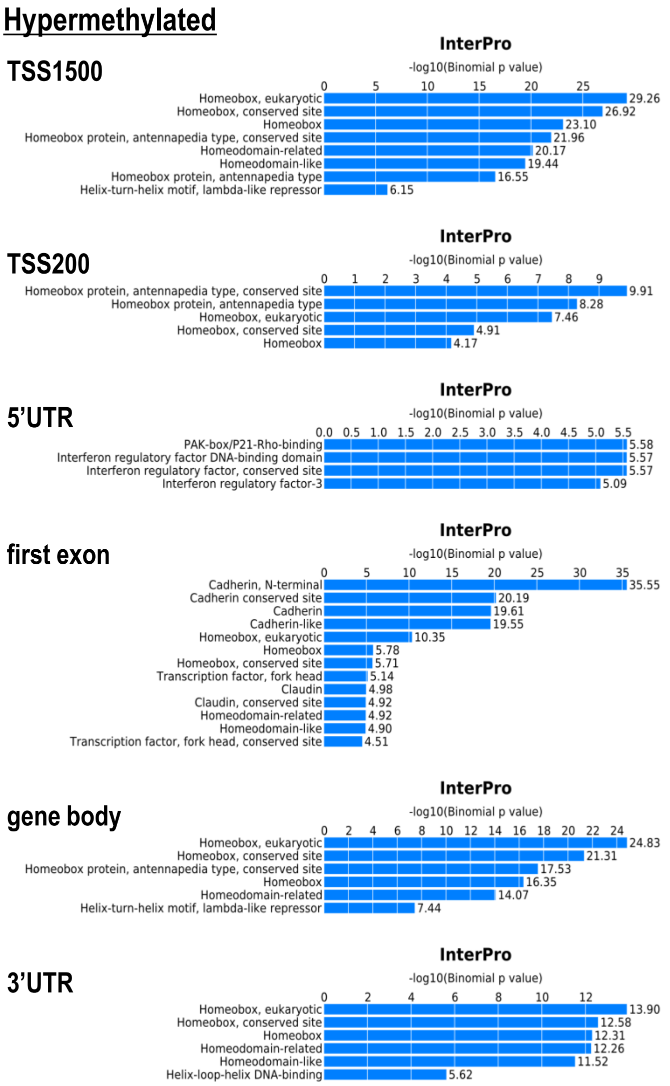

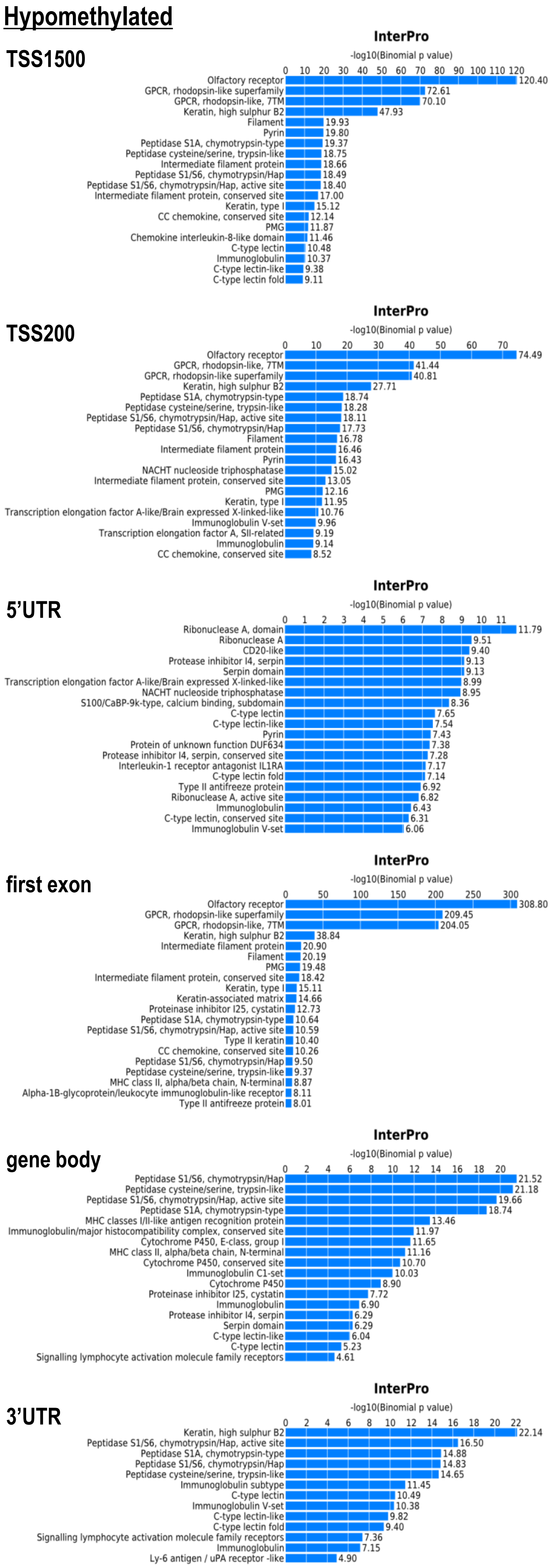

Supplementary Figure S3: Ontology terms in the InterPro category detected to be enriched among the differentially methylated regions in LMS (compared to NM) in the GREAT annotation. Blue bar charts represent binomial *P*-values (in -log10 scale) indicating statistical significance.

Supplementary Figure S4: DNA methylation profiles of NM, LM, and LMS samples visualized using Integrative Genomic Viewer (https://www.broadinstitute.org/igv/).

β values of NM (average of three samples), individual LM and LMS samples are shown in light blue, blue, and purple vertical bars, respectively, together with β values of six tissues from healthy individuals (obtained from GEO GSE52578 and GSE36369/GSM1023114) shown in black vertical bars. Hypermethylation and hypomethylatin blocks defined as cancer-specific differentially DNA-methylated regions (cDMRs) in a previous epigenomic study for colon cancers [30] are shown by red and green horizontal bars as "hypermethylated cDMRs" and "hypomethylated cDMRs". **(a)** The *HOXA* gene cluster hypermethylated in both LM and LMS samples. **(b)** The *PITX2* gene locus hypermethylated in LMS samples. **(c)** A large hypomethylated block in LMS at the *KRTAP10* gene cluster on chr.21 **(d)** Large hypomethylated blocks in LMS at 19q13.33-13.42 region. In the four loci presented, it is consistently observed that while the DNA methylation profiles of three LM samples are similar overall, those of three LMS samples are locally variable. This LMS-specific epigenomic feature coincides well with the previously reported stochastic methylation variation of cancer-specific differentially DNA-methylated regions observed in several cancers [30].

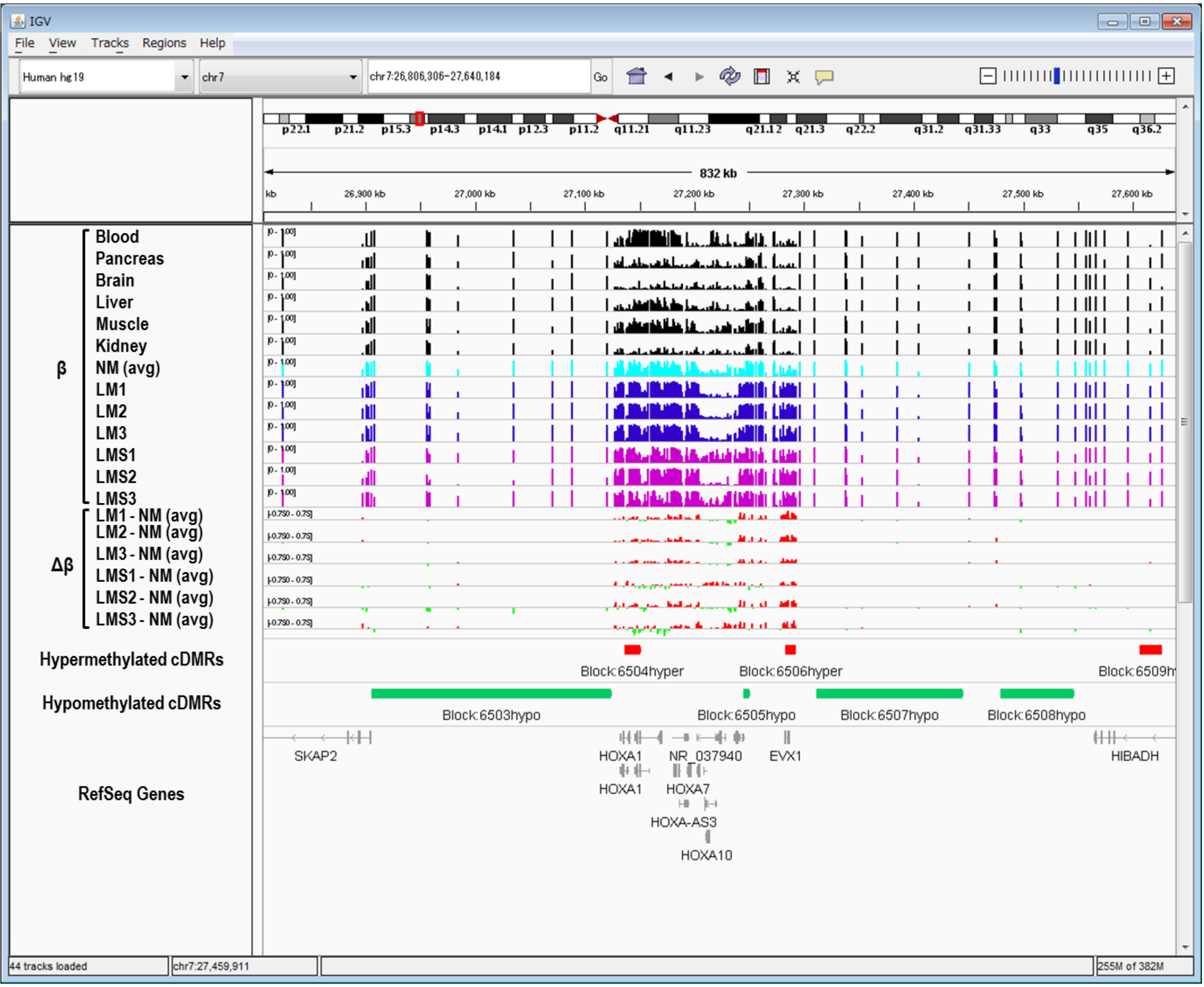

Supplementary Figure S4(a): The *HOXA* gene cluster hypermethylated in both LM and LMS samples

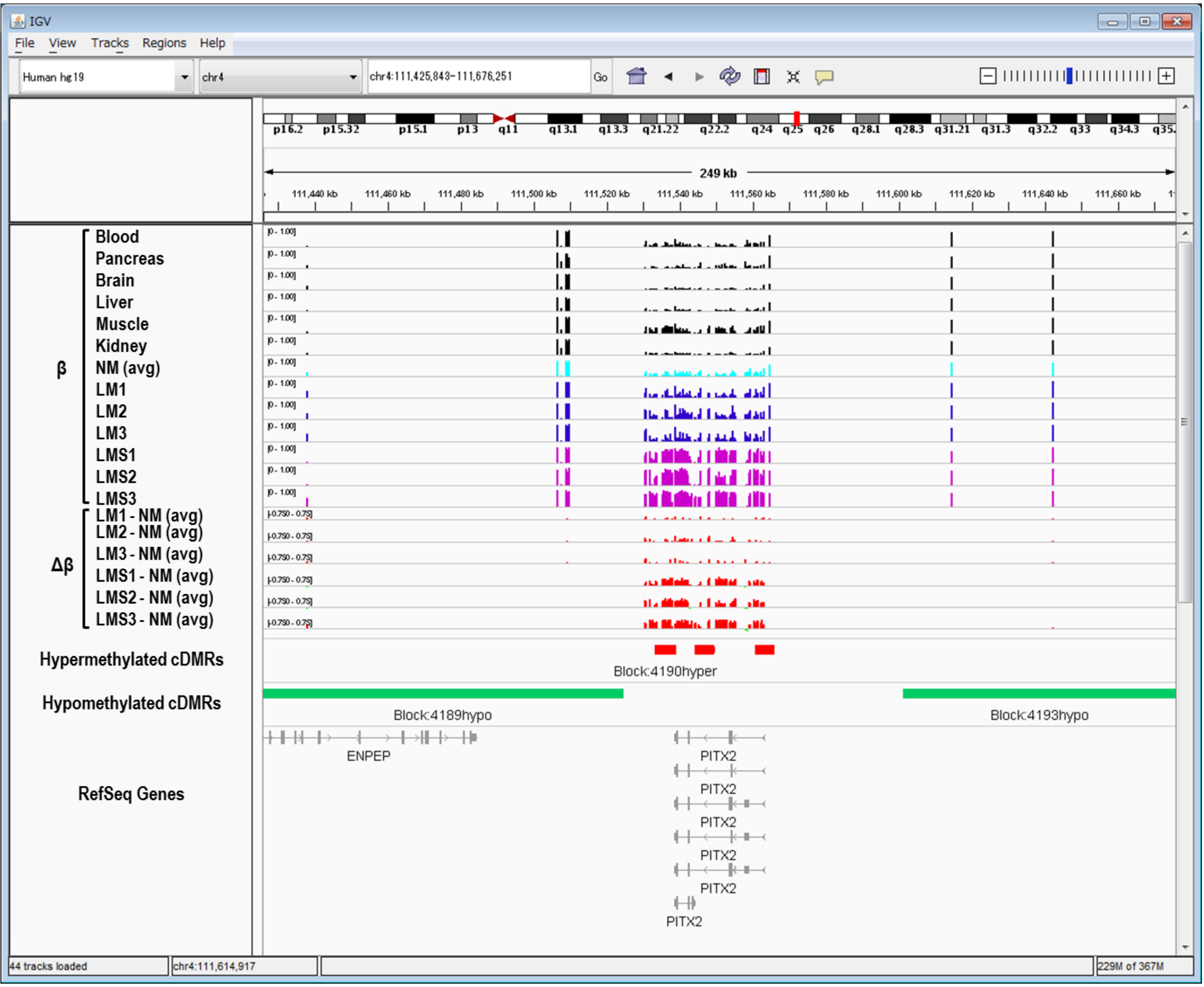

Supplementary Figure S4(b): The *PITX2* gene locus hypermethylated in LMS samples

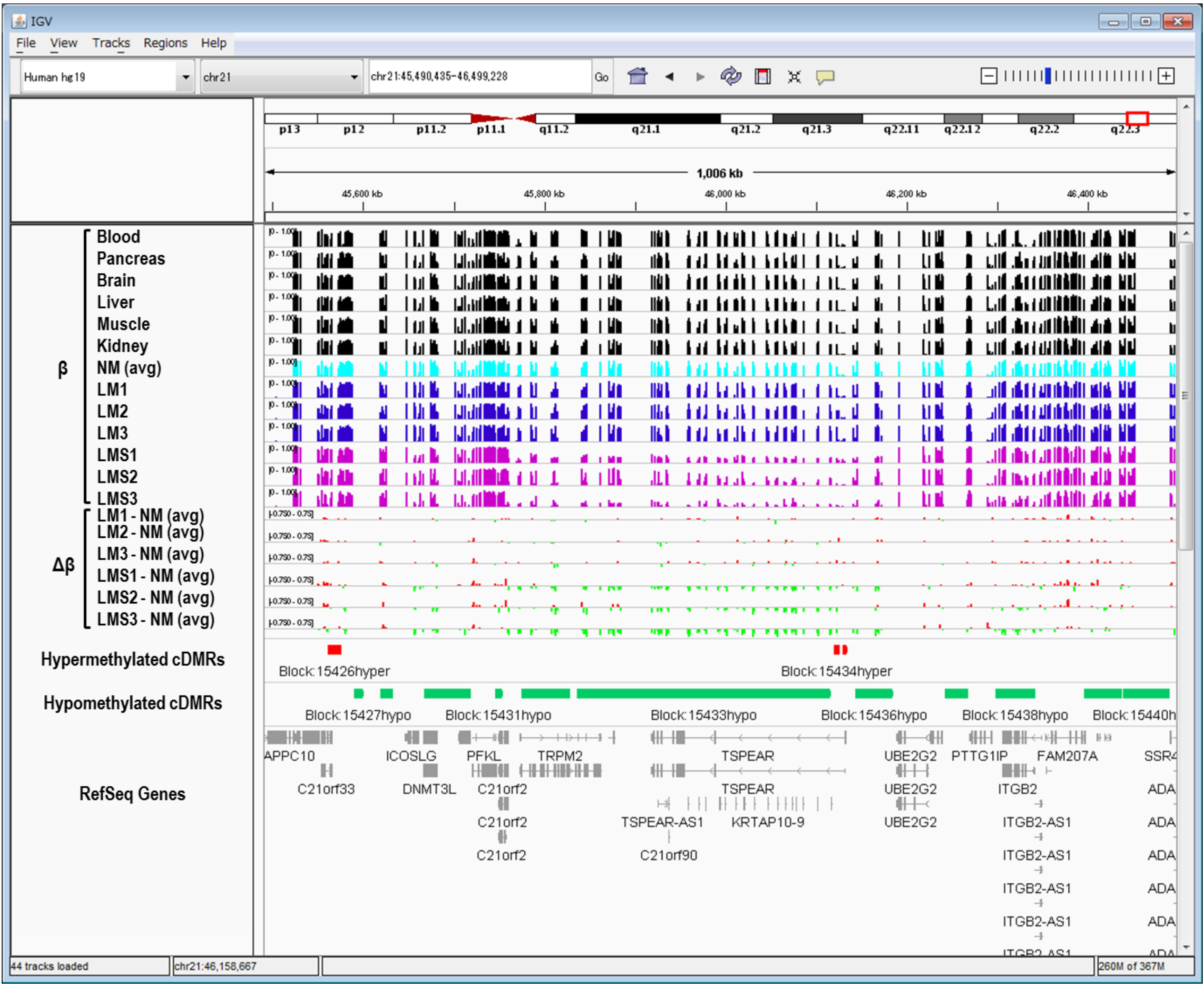

Supplementary Figure S4(c): A large hypomethylated block in LMS at the *KRTAP10* gene cluster on chr.21

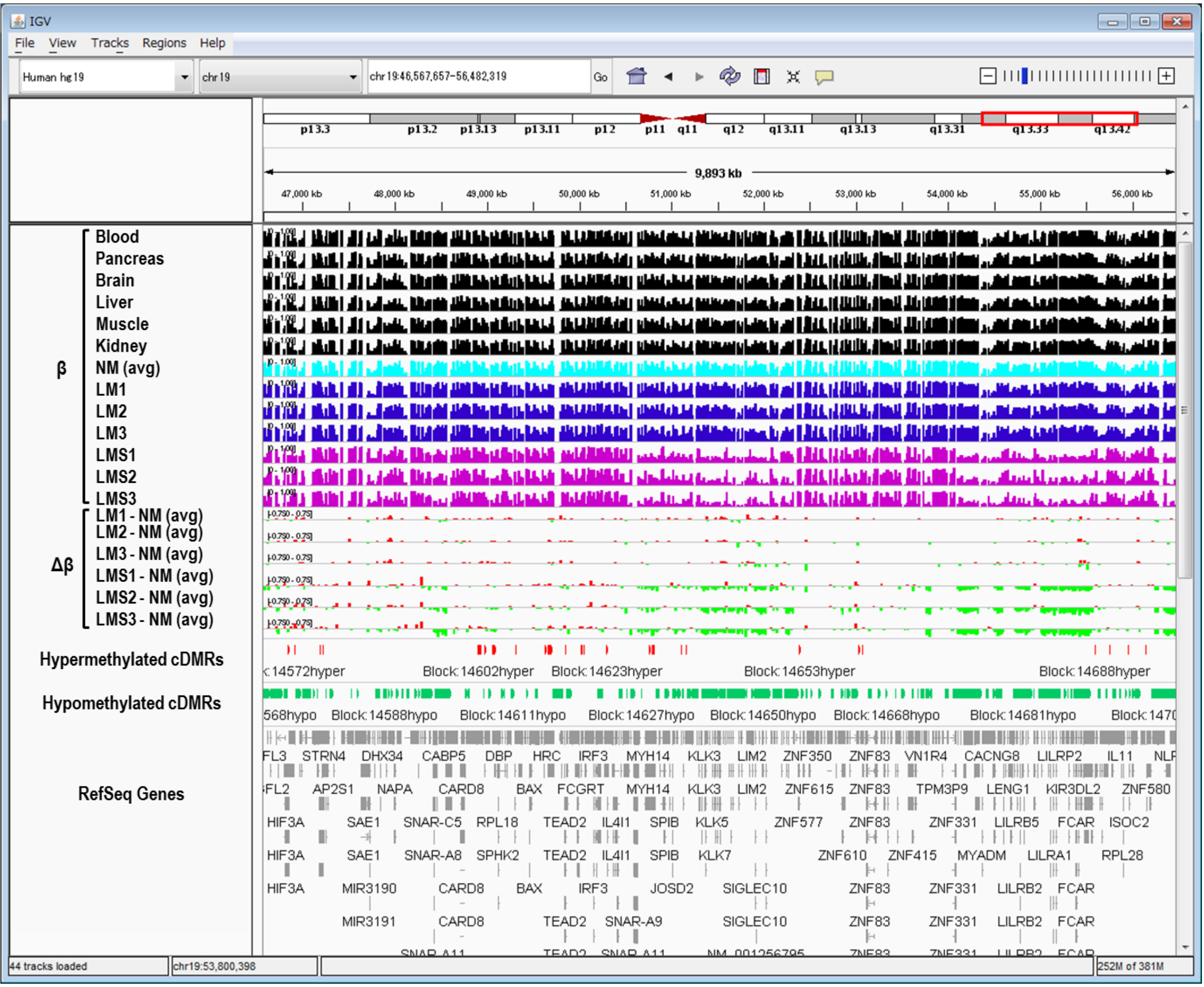

Supplementary Figure S4(d): Large hypomethylated blocks in LMS at 19q13.33-13.42 region

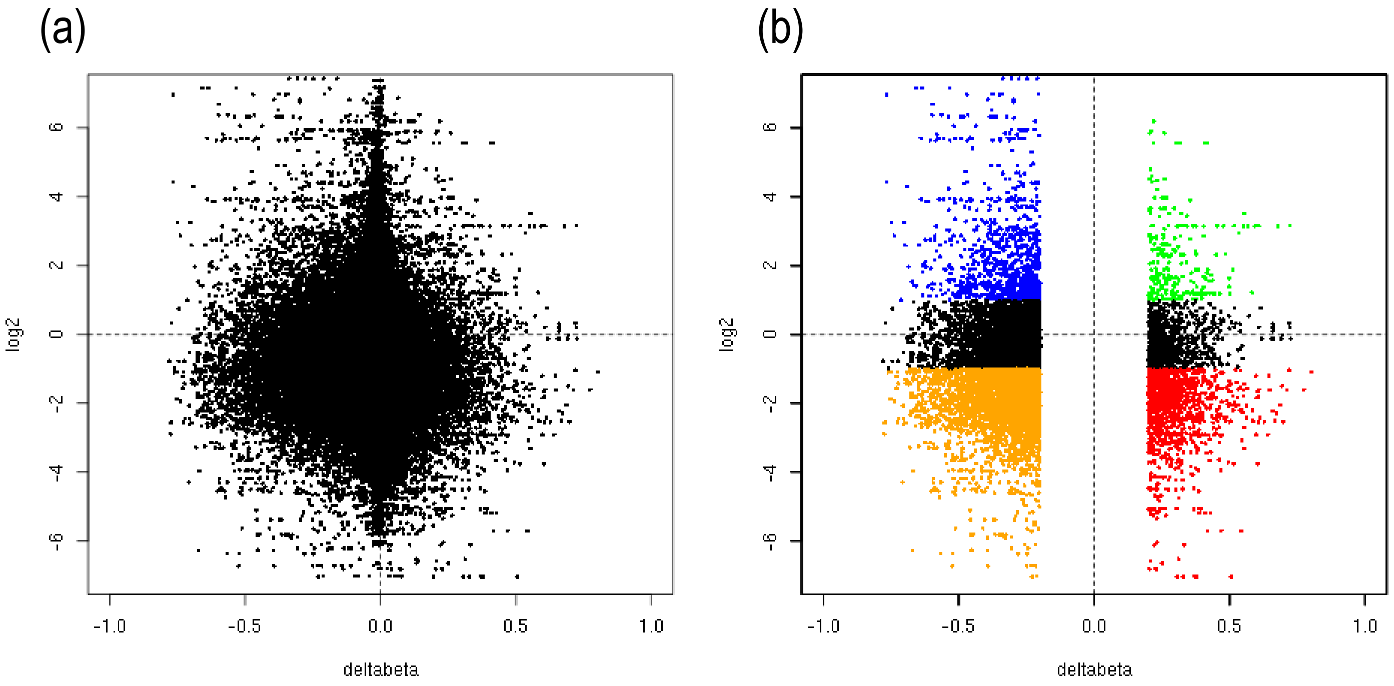

Supplementary Figure S5:

Methylation by expression plots of LMS compared to NM.

HumanMethylation450 probes within TSS200 and TSS1500 regions (121,066 probes) were plotted for their differential methylation levels (Δβ values, x-axis) and for the differential expression levels of the corresponding 14,236 GenBank mRNA accession numbers (log2-transformed fold-change values, y-axis) in LMS compared to NM. The average values of three samples each of LMS and NM were compared.

(a) The methylation by expression plot for all probes (121,066 probes vs 14,236 mRNA accession numbers). Correlation coefficient, -0.019.

(b) The methylation by expression plot for the probes whose Δβ value is > 0.2 or < -0.2 (10,492 probes). The numbers of the probes shown in blue (Δβ < -0.2 and log2 > 1), orange (Δβ < -0.2 and log2 < -1), red (Δβ > 0.2 and log2 < -1), green (Δβ > 0.2 and log2 > 1) and black were 989, 3582, 1101, 238 and 4582, respectively. Correlation coefficient for the probes in blue, orange, red, and green regions (5910 probes in total), -0.088.
