# Supplementary material for: Genomic, Epigenomic, and Transcriptomic Profiling towards Identifying Omics Features and Specific Biomarkers That Distinguish Uterine Leiomyosarcoma and Leiomyoma at Molecular Levels
Source: Sarcoma. 2015 Dec 28;2015:412068. doi: 10.1155/2015/412068 (PMC4707342; doi:10.1155/2015/412068)
Supplement: Supplementary file 4 [file 412068.f4.pdf]

Table S3: full list of candidate expression markers selected using filter conditions shown in Table S2

Type	Normalized log2 intensity												Gene Symbol	ProbeName	Description	RefSeq Accession
	NM1	NM2	NM3	LM1	LM2	LM3	LMS1	LMS2	LMS3	SK-UT1	SK-UT1B	SKN				
NMLM-specific	12.8	13.2	12.5	13.9	13.7	15.0	3.1	2.7	2.3	2.7	2.7	2.8	*PGR*	A_32_P49199	Homo sapiens progesterone receptor (PGR), transcript variant 2, mRNA	NM_000926
	9.3	12.0	8.9	7.4	7.5	9.7	3.3	3.3	3.2	3.0	2.8	4.1	*ACKR1*	A_23_P115161	Homo sapiens atypical chemokine receptor 1 (Duffy blood group) (ACKR1), transcript variant 2, mRNA	NM_002036
	10.0	6.8	8.4	8.8	6.7	7.8	3.3	2.8	2.4	2.6	2.6	2.7	*DPP6*	A_24_P6552	Homo sapiens dipeptidyl-peptidase 6 (DPP6), transcript variant 3, mRNA	NM_001039350
	7.8	8.6	7.5	7.7	6.4	8.7	2.5	2.5	2.5	2.4	2.4	2.4	*RIC3*	A_24_P35537	Homo sapiens RIC3 acetylcholine receptor chaperone (RIC3), transcript variant 1, mRNA	NM_024557
NM-specific	6.3	10.5	9.4	2.8	3.4	6.4	3.2	2.6	2.3	2.8	2.8	2.8	*CYP4B1*	A_23_P114713	Homo sapiens cytochrome P450, family 4, subfamily B, polypeptide 1 (CYP4B1), transcript variant 2, mRNA	NM_000779
	6.6	9.6	8.1	3.3	2.6	6.7	3.9	2.7	2.7	2.8	2.7	3.0	*STEAP4*	A_24_P351906	Homo sapiens STEAP family member 4 (STEAP4), transcript variant 1, mRNA	NM_024636
	8.4	7.6	7.6	4.1	5.3	3.1	3.6	3.9	3.6	2.6	3.0	3.4	*MS4A2*	A_23_P1904	Homo sapiens membrane-spanning 4-domains, subfamily A, member 2 (MS4A2), transcript variant 1, mRNA	NM_000139
	7.1	7.8	8.2	4.5	4.9	3.1	2.8	2.6	2.6	2.9	3.5	3.1	*LCN6*	A_23_P83184	Homo sapiens lipocalin 6 (LCN6), mRNA	NM_198946
LM-specific	4.6	4.3	2.2	10.3	9.8	8.2	4.0	4.0	3.0	3.0	2.9	3.2	*SMPX*	A_23_P253542	Homo sapiens small muscle protein, X-linked (SMPX), transcript variant 1, mRNA	NM_014332
LMS-specific	3.9	3.4	3.7	2.7	3.2	3.1	10.5	12.0	9.8	10.1	10.8	12.9	***FOXD1***	A_32_P34920	Homo sapiens forkhead box D1 (FOXD1), mRNA	NM_004472
	4.9	3.3	4.1	3.5	4.7	3.1	11.6	9.7	10.6	13.4	12.9	12.4	***CDCA2***	A_23_P385861	Homo sapiens cell division cycle associated 2 (CDCA2), mRNA	NM_152562
	2.7	3.0	2.3	4.3	4.8	5.1	11.1	9.7	10.1	12.4	12.3	11.5	***MELK***	A_23_P94422	Homo sapiens maternal embryonic leucine zipper kinase (MELK), transcript variant 1, mRNA	NM_014791
	2.9	2.5	2.8	3.9	3.9	3.1	10.9	9.3	10.5	12.7	13.1	11.9	***TICRR***	A_32_P109296	Homo sapiens TOPBP1-interacting checkpoint and replication regulator (TICRR), mRNA	NM_152259
	2.5	2.9	2.2	3.3	2.5	3.1	11.1	9.5	9.7	11.6	11.4	11.1	***KIF4A***	A_23_P148475	Homo sapiens kinesin family member 4A (KIF4A), mRNA	NM_012310
	2.8	2.8	2.5	4.4	2.3	3.1	9.4	9.5	11.0	9.2	9.5	7.7	*GPR158*	A_24_P349117	Homo sapiens G protein-coupled receptor 158 (GPR158), mRNA	NM_020752
	2.7	4.0	4.4	3.4	3.6	4.9	10.2	8.9	9.3	11.0	10.7	9.3	*MTFR2*	A_23_P253752	Homo sapiens mitochondrial fission regulator 2 (MTFR2), transcript variant 2, mRNA	NM_138419
	2.3	4.0	2.4	2.9	2.9	3.1	10.3	8.9	9.0	10.7	10.0	10.4	*KIF18A*	A_23_P150667	Homo sapiens kinesin family member 18A (KIF18A), mRNA	NM_031217
	3.7	2.9	2.5	2.3	4.1	3.1	9.6	9.3	9.3	10.8	10.3	10.1	***TTK***	A_23_P259586	Homo sapiens TTK protein kinase (TTK), transcript variant 1, mRNA	NM_003318
	2.8	2.5	3.3	3.5	3.3	3.1	9.7	7.8	10.2	10.5	11.0	10.1	*ESCO2*	A_24_P323598	Homo sapiens establishment of sister chromatid cohesion N-acetyltransferase 2 (ESCO2), mRNA	NM_001017420
	3.0	2.5	2.7	3.3	3.1	3.1	9.4	8.6	9.4	10.1	9.5	9.7	*AURKB*	A_23_P130182	Homo sapiens aurora kinase B (AURKB), transcript variant 1, mRNA	NM_004217
	2.3	2.5	2.6	2.5	2.3	3.1	9.8	8.0	9.3	10.8	10.7	10.5	*CENPA*	A_24_P413884	Homo sapiens centromere protein A (CENPA), transcript variant 1, mRNA	NM_001809
	4.2	3.0	2.5	4.5	4.7	3.1	10.0	9.0	7.9	11.0	10.7	11.1	*ARHGAP11A*	A_23_P136805	Homo sapiens Rho GTPase activating protein 11A (ARHGAP11A), transcript variant 1, mRNA	NM_014783
	3.2	2.5	2.4	2.5	2.6	3.1	10.2	8.2	8.4	11.0	10.6	10.6	*TRMU*	A_24_P916195	tRNA 5-methylaminomethyl-2-thiouridylate methyltransferase	XM_005261627
	2.8	3.1	2.7	3.3	3.7	5.1	9.5	8.1	8.7	10.9	10.8	10.3	*CCNA2*	A_23_P58321	Homo sapiens cyclin A2 (CCNA2), mRNA	NM_001237
	2.3	2.5	2.4	4.3	2.9	5.7	9.0	8.9	8.2	11.0	11.0	9.4	*POLQ*	A_23_P218827	Homo sapiens polymerase (DNA directed), theta (POLQ), mRNA	NM_199420
	2.7	2.5	2.3	3.8	3.0	3.1	9.5	8.5	8.0	10.1	10.5	9.7	*CENPM*	A_24_P399888	Homo sapiens centromere protein M (CENPM), transcript variant 2, mRNA	NM_001002876
	2.5	2.5	2.2	4.4	2.6	5.8	10.0	8.5	7.4	12.1	12.4	11.1	*RRM2*	A_24_P225616	Homo sapiens ribonucleotide reductase M2 (RRM2), transcript variant 2, mRNA	NM_001034
	3.7	3.9	2.6	2.5	3.3	3.1	9.3	8.5	8.0	11.1	11.3	10.8	*KIF18B*	A_24_P680947	Homo sapiens kinesin family member 18B (KIF18B), transcript variant 2, mRNA	NM_001264573
	2.3	2.5	2.5	3.3	2.4	3.1	10.0	8.0	7.8	10.8	11.5	10.3	*MCM10*	A_23_P161474	Homo sapiens minichromosome maintenance complex component 10 (MCM10), transcript variant 1, mRNA	NM_182751
	4.6	4.9	2.4	4.2	2.2	3.1	8.6	8.6	8.4	9.7	9.7	9.6	*CENPN*	A_23_P355075	Homo sapiens centromere protein N (CENPN), transcript variant 2, mRNA	NM_001100624
	3.2	2.5	2.4	2.5	2.5	3.1	7.3	8.4	9.8	9.7	9.9	9.3	*MKI67*	A_24_P346855	Homo sapiens marker of proliferation Ki-67 (MKI67), transcript variant 1, mRNA	NM_002417
	2.5	2.5	2.5	4.0	3.0	7.1	6.4	7.3	11.4	10.9	11.0	8.0	*LHX2*	A_23_P32165	Homo sapiens LIM homeobox 2 (LHX2), mRNA	NM_004789
	2.4	2.5	2.8	3.2	4.7	6.4	9.3	8.3	7.4	9.5	9.5	9.5	*SGOL1*	A_24_P225970	Homo sapiens shugoshin-like 1 (S. pombe) (SGOL1), transcript variant A1, mRNA	NM_001012409
	2.9	2.9	3.2	2.6	4.3	3.1	9.2	8.4	7.4	9.1	8.4	8.5	*ASPM*	A_24_P911179	Homo sapiens asp (abnormal spindle) homolog, microcephaly associated (Drosophila) (ASPM), transcript variant 1, mRNA	NM_018136
	2.4	2.5	2.5	2.8	3.1	5.4	9.0	7.9	8.1	11.2	11.0	10.9	*HJURP*	A_24_P257099	Homo sapiens Holliday junction recognition protein (HJURP), transcript variant 1, mRNA	NM_018410
	2.4	5.7	3.0	3.9	4.3	4.7	9.4	7.8	7.6	10.4	11.2	9.7	*MCM10*	A_24_P412088	Homo sapiens minichromosome maintenance complex component 10 (MCM10), transcript variant 1, mRNA	NM_182751
	4.1	2.5	2.4	4.5	2.9	3.1	8.2	7.7	8.7	9.7	9.5	8.9	*PLK4*	A_23_P155969	Homo sapiens polo-like kinase 4 (PLK4), transcript variant 1, mRNA	NM_014264
	2.6	3.1	4.5	3.9	3.4	5.7	9.3	8.4	6.8	11.0	10.6	9.9	*PLK1*	A_23_P118174	Homo sapiens polo-like kinase 1 (PLK1), mRNA	NM_005030
	2.4	5.7	2.9	3.6	3.3	6.6	8.3	8.1	7.9	11.2	11.7	10.8	*SAPCD2*	A_24_P100517	Homo sapiens suppressor APC domain containing 2 (SAPCD2), mRNA	NM_178448
	3.0	3.5	3.2	2.5	2.7	3.1	8.1	7.4	8.0	9.1	8.9	8.3	*NEIL3*	A_23_P155711	Homo sapiens nei endonuclease VIII-like 3 (E. coli) (NEIL3), mRNA	NM_018248
	2.3	2.5	2.4	3.3	2.4	3.1	9.1	6.8	7.1	9.1	9.2	7.6	*STRIP2*	A_32_P233735	Homo sapiens striatin interacting protein 2 (STRIP2), transcript variant 1, mRNA	NM_020704
	4.0	2.5	2.4	3.4	2.5	5.2	9.8	6.7	6.1	12.2	7.7	10.1	*RAB3B*	A_24_P933319	Homo sapiens RAB3B, member RAS oncogene family (RAB3B), mRNA	NM_002867
	3.6	3.7	2.8	4.4	3.9	4.9	6.3	8.3	7.8	9.4	8.9	9.3	*FBXL5*	A_32_P108748	F-box and leucine-rich repeat protein 5	XR_425710
	3.7	2.5	2.6	2.3	2.6	3.1	8.0	7.0	6.8	10.0	9.3	9.4	*CDCA2*	A_24_P323434	Homo sapiens cell division cycle associated 2 (CDCA2), mRNA	NM_152562
	2.4	2.5	2.5	4.2	2.5	3.1	8.3	6.1	7.4	8.6	8.6	8.4	*GINS4*	A_23_P136786	Homo sapiens GINS complex subunit 4 (Sld5 homolog) (GINS4), mRNA	NM_032336
	2.6	4.7	3.8	3.4	4.5	4.8	6.9	6.6	7.9	5.6	6.7	9.6	*ADAMTS14*	A_24_P275073	Homo sapiens ADAM metallopeptidase with thrombospondin type 1 motif, 14 (ADAMTS14), transcript variant 1, mRNA	NM_139155
	4.6	2.5	2.5	4.2	2.4	3.1	7.3	6.8	7.0	9.6	10.1	9.2	*FANCD2*	A_23_P143994	Homo sapiens Fanconi anemia, complementation group D2 (FANCD2), transcript variant 2, mRNA	NM_001018115
	3.5	2.5	2.4	2.7	2.3	3.1	7.3	7.2	6.6	9.3	8.4	8.0	*NEK2*	A_24_P319613	Homo sapiens NIMA-related kinase 2 (NEK2), transcript variant 1, mRNA	NM_002497
	3.3	2.5	2.5	4.1	2.7	3.1	6.1	6.4	8.5	6.6	6.5	5.8	*SYCE2*	A_32_P203528	Homo sapiens synaptonemal complex central element protein 2 (SYCE2), mRNA	NM_001105578
	3.9	2.5	2.5	4.4	2.8	3.1	7.4	6.3	7.1	8.4	8.2	7.5	*RELT*	A_23_P47735	Homo sapiens RELT tumor necrosis factor receptor (RELT), transcript variant 1, mRNA	NM_032871
	3.1	2.5	2.5	4.1	2.4	3.1	7.7	7.0	6.1	9.3	9.8	8.1	*ORC1*	A_23_P45799	Homo sapiens origin recognition complex, subunit 1 (ORC1), transcript variant 1, mRNA	NM_004153
	2.5	2.8	2.3	2.9	2.7	3.1	6.4	7.3	6.9	8.5	8.7	7.8	*GSG2*	A_23_P66732	Homo sapiens germ cell associated 2 (haspin) (GSG2), mRNA	NM_031965
	2.3	2.5	2.9	3.9	2.5	3.1	6.8	6.9	6.7	9.3	9.3	8.3	*CCNF*	A_23_P37954	Homo sapiens cyclin F (CCNF), mRNA	NM_001761

LMS-specifc genes shown in Fig.2C are shown in red.

Normalized log2 intensity: 2.0, 3.0, 4.0, 5.0, 6.0, 7.0, 8.0, 9.0, 10.0, 11.0, 12.0, 13.0, 14.0
